# Influence of HLA-B*5701 on 20 year survival rate among patients living with HIV

**DOI:** 10.1371/journal.pone.0255834

**Published:** 2021-08-09

**Authors:** Bogusz Jan Aksak-Wąs, Miłosz Parczewski, Anna Urbańska, Małgorzata Hackiewicz, Justyna D. Kowalska

**Affiliations:** 1 Department of Infectious, Tropical Diseases and Immune Deficiency, Pomeranian Medical University in Szczecin, Szczecin, Poland; 2 Department of Adults’ Infectious Diseases, Medical University of Warsaw, Warsaw, Poland; University of Ghana College of Health Sciences, GHANA

## Abstract

**Background:**

The life expectancy of people living with HIV (PLWH) remains shorter than that of the general population, despite significant improvement in the recent years. Mortality in HIV-infected individuals may be associated with a higher viral load at of diagnosis, a lower CD4 count, or clinical variables such as sex or route of transmission. This article investigated the role of the HLA-B*5701 varian on mortality among PLWH.

**Methods:**

Material for the analysis consist of the data of 2,393 patients for whom the HLA-B*57 variant was known. Those patients were followed under the care of the Infectious Diseases Hospital in Warsaw (n = 1555) and the Clinic of Acquired Immunodeficiency of the Pomeranian Medical University in Szczecin (n = 838). Factors such as age, gender, date of HIV diagnosis, route of transmission, date of death, baseline HIV viral load and baseline CD4 counts, were collected, and end-point cross-sectional analyses were marked at 60, 120, 180 and 240 month of observation.

**Results:**

HLA-B*5701 allele was found in 133 (5.5%) analyzed cases. Median age was notably higher for HLA-B*5701 positive patients [32.7 (28.3–41.3) vs. 31.6 (26.8–38.3)years p = 0.02]. HLA-B*5701 was associated with lower baseline viral load [4.21 (3.5–4.8) vs. 4.79 (4.2–5.3)log copies/ml p<0.001] and higher CD4count [448 (294.5–662) vs. 352 (176–514) cells/μl p<0.001]. There were no association between HLA-B*5701 and survival for any given end-point. Higher mortality was associated to male gender, intravenous drug users, lower CD4 count at baseline and higher baseline viral load.

**Conclusions:**

In our study, the presence of HLA-B*5701 allel was not associated with mortality rate of HIV infected patients, irrespective of being associated with both higher baseline CD4 + cell count and lower baseline HIV viral load.

## Introduction

Despite notable survival disparities in the early HIV epidemics, following the introduction of combined antiretroviral therapy (cART) the life span of people living with HIV (PLWH) is currently approaching the life expectancy of HIV uninfected individuals. Depending on region and gender it may range from 60.3% (mean: 37.4 years) in Rwanda to 89.1% in Canada (mean: 72.3 years) compared to the HIV-uninfected population [1].

Mortality risk remains associated with the delayed HIV diagnosis, despite observed progress in the cART efficacy and widespread implementation of the CD4 count threshold unrestricted initiation schemes [2–4]. Nowadays, the global consensus is to treat every person living with HIV as soon as feasible, irrespective of the CD4 count, as stipulated by international and national treatment guidelines [5–7]. Survival remains dependent on the presence of the array of risk factors, which invariably include AIDS-defining illnesses, malignancy or suicide [8].

Alongside with these key factors affecting HIV mortality, a wide array of genetic factors may also influence survival in this group mostly by affecting HIV viral load or the tempo of lymphocyte CD4 decay. One of the examples includes the single nucleotide polymorphism (rs9264942) located -35 k.b. upstream of the HLA-C gene. Frequency of variant genotypes vary notably in European populations (from 17.6% [9] to 29.8% [10]) with ~90% viral load reduction in antiretroviral untreated individuals observed in CC homozygotes [11].

Other well studied gene variants include chemokine receptor 5 (CCR5) located on 3^rd^ chromosome (position p21)—including 32 base pair mutation (Δ32) in the coding region of this gene associated with the slower HIV disease progression and positive influence on the survival among cART untreated patients [12]. This deletion in the CCR5 gene is present in 5–14% of European citizens but is virtually absent among cases of Asian and African ancestry [13].

Additionally, an array of HLA variants such as HLA-Cw0102, HLA-B*5701 and HLA-B*2705, as well as allele combinations like HLA-B*5701-Cw0602, HLA-B*2705-Cw0102, or HLA-B*3801-Cw1203 were associated with modification of the HIV viral loads, differences in the tempo of the lymphocyte CD4 depletion [14] and may affect the survival among PLWH. Of these one of the variants widely associated with significant protective effect on the HIV replication and delayed disease progression is HLA-B*5701 [15]. It was confirmed that HLA B*5701 allele is more common among slowly progressing individuals and HIV elite controllers [15]. This split antigen of HLA B17 and a pharmacogenetic marker of abacavir induced hypersensitivity is widely implemented in the clinical practice related to HIV. Prior to the routine introduction of the HLA-B*5701 testing approximately 8% of patients receiving abacavir presented with hypersensitivity reactions (including the risk of life-threatening anaphylactic reaction in the cases of re-challenge with the drug) [16]. Introduction of the pharmacogenomic testing for this allele allowed to reduce this risk with the positive prediction value (PPV) of 61.2%, negative prediction value (NPV) of 95.5%, sensitivity of 44% and 96% specificity [17]. Prevalence of this genetic variant varies from 1.5% to 7.8%, with mean frequency of ~5% in Europe [9, 18, 19]. Presence of the HLA B*5701 was associated with beneficial clinical characteristics and lower HIV viral loads especially among individuals with presence of other beneficial genetic variants such as HLA-C-35 CC genotype [9]. Having already associated the presence of HLA B*5701 with lower viral load in our observational cohorts, we wished to study its influence on the long-term survival in association with clinical, virologic and immunologic characteristics over the longer periods of follow-up defined as 20 years of observation.

## Materials and methods

### Study population

For the study longitudinal data of 2393 patients with available HLA-B*5701 genotype followed-up at the Regional Infectious Diseases Hospital in Warsaw (n = 1555), Poland and Department of Acquired Immunodeficiency, Pomeranian Medical University in Szczecin (n = 838) were collected. The study protocol was approved by the bioethical committee of Pomeranian Medical University, Szczecin, Poland (approval number BN-001/34/04). Written, informed consent was obtained from all subjects participating in the study. All data were fully anonymized before statistical analyses.

The following data were collected: age, gender, date of HIV diagnosis, route of transmission, date of death, baseline HIV viral load and baseline CD4 counts. Baseline CD4 counts were defined as the first documented result after diagnosis of HIV.

### Observation time and the study endpoints

Observation time was defined as a period from time zero (date of positive screening HIV test if later confirmed by Western-blot, immunoblotting or serum HIV-RNA) to the timepoint of the end of observation defined as either death date, last recorded date of visit (cases lost to follow-up) or 31 January 2019 for the patients remaining under care (termination of data collection). For pretreatment survival analyses the endpoint was cART initiation date, while for survival analyses on cART initiation of observation period was defined as the date of antiretroviral treatment introduction, with the endpoint set as the database closure. In cases when it was impossible to determine the exact date of death the median date between the last recorded visit and information on death was assumed as the death date. For this study all-cause mortality without any exclusions was analysed. Determination of the reason of death was based on the following data: autopsy report, medical record of in-hospital treatment with cause of death defined by the treating physician, other medical report or letter.

### DNA extraction and HLA genotyping

QIAamp DNA Blood Mini Kit (QIAgen, Hilden, Germany) was used to extract genomic DNA from samples previously collected into tubes containing EDTA anticoagulant. The extraction was performed following to the manufacturer’s protocol, DNA was re-suspended in 200 μL of AE buffer (QIAgen, Hilden, Germany) and stored at 4°C for further analyses. HLA-B*5701 screening was performed using SSP HLA-Ready gene B5/57 cross low resolution kit (Inno-Train Diagnostik, Kronberg, Germany) by a in vitro diagnostics validated, CE marked test, according to the manufacturer’s protocol. PCR products were electrophoresised on a 3%, agarose gel (SIGMA, Saint Louis, USA) stained with Gel-Star dye (Lonza, Switzerland). Results were visualized under UV light (Transilluminator 4000, Stratagene, La Jolla, USA) and recorded with DS-34 Polaroid Direct Screen Camera. Additionally, all HLA-B*5701 positive samples were verified using another CE marked assay—Olerup SSP HLA-B*57 high resolution kit (Olerup SSP AB, Saltsjoebaden, Sweden), with subsequent electrophoresis and recording as described above.

CD4+ lymphocyte count was obtained during flow cytometry analysis for every patient at a beginning of observation (baseline or pretreatment count). HIV RNA viral load was obtained from plasma samples routinely taken from patients attending corresponding clinics.

### Statistics

Statistical analyses for nominal variables were performed with Chi-square test (gender, HIV infection stage at genotyping, transmission route), while for continuous variables (T CD4+ lymphocyte count, HIV viral load at care entry) the Mann–Whitney U-test was used (Statistica v. 13 software, Statsoft, Poland). Kaplan-Meyer cumulative mortality was calculated for the analysed dataset, survival data were analyzed using log-rank test for 60, 120, 180 and 240 month end point. Unaivariate and multivariate Cox proportional hazards models were used to assess the effect of analyzed parameter on the risk of death and to calculate the hazard ratios (HR). For survival statistics the men who have sex with men and heterosexual categories were joint into one category. P-values of 0.05 were considered significant and confidence intervals (CIs) of 95% were accepted.

## Results

### Cohort characteristics

HLA-B*5701 allele was found in 133 (5.5%) analysed cases (Table 1). For the individuals included in the study, median observation time was 43.2 (IQR: 17.2–101.5) months, median time on cART was 34.6 months (IQR: 14.1–78.6). Median time from diagnosis to the cART initiation was 2.8 (IQR: 0–21.5) months. In total 2125 (88,8%) patients received cART (105 (78,9%) in the HLA-B*5701 positive and 2182 (96,5%) among the HLA-B*5701 negative cases (**p<0.001**). Undetectable viral load in 12 months after starting treatement was obtained in 1570 cases (77in the HLA-B*5701 positive and 1493 among the HLA-B*5701 negative cases.

**Table 1:** Baseline characteristics in examined cohort.

	HLA-B*5701 (n = 2393)			
	positive	negative	p value	total
***Gender*, *n (%)***				
Female	27 (20.3)	399 (17.6)	p = 0.4	426 (17.8)
Male	106 (79.7)	1861 (82.3)		1967 (82.2)
**Age at diagnosis, median (IQR) years**	32.7 (28.3–41.3)	31.6 (26.8–38.3)	p = 0.02	31.7 (26.8–38.6)
***Route of transmission*** * **, *n (%)***				
Men who have sex with men	52 (49.5)	935 (54.5)	p = 0.6	987 (54.3%)
Heterosexual	30 (28.6)	431 (25.1)		461 (25.3%)
People who inject drugs	23 (21.9)	348 (20.3)		371 (20.4%)
	105	1714		
***Baseline lymphocyte CD4 count*, *median (IQR) cells/μl***	448 (294.5–662)	352 (176–514)	p<0.001	354 (181–517)
***Baseline lymphocyte CD4 count <200 cells/μl*, *n (%)***	16 (12.5)	609 (28.2)	p<0.001	625 (27.4)
***Baseline lymphocyte CD4 count >200 cells/μl*, *n (%)***	112 (87,5)	1548 (71,8)		1660 (72,6)
***Baseline viral load characteristics***				
***Baseline HIV-1 viral load*, *median (IQR) log copies/ml***	4.21 (3.5–4.8)	4.79 (4.2–5.3)	p<0.001	4.77 (4.15–5.28)
***VL at diagnosis <5 log copies/ml*, *n (%)***	97 (82.9)	1228 (61.3)	p<0.001	1325 (62.5)
***VL at diagnosis >5 log copies/ml*, *n (%)***	20 (17.1)	776 (38.7)		796 (37.5)

*transmission route available for 1819 cases, Lymphocyte CD4 count for 2285, HIV-1 baseline viral load for 2121 cases.

There were no significant differences in the time from diagnosis to cART initiation in HLA-B*5701 positive and negative patients (15,2 (IQR: 0–68,3) months vs 14,8 (IQR: 0–63,3) months, p = 0.74). In total, 157 (6.4%) deaths were observed in examined cohort including 4 (3.0%) cases among HLA-B*5701 positive patients and 153 (7.3%) in HLA-B*5701 negative group, p = 0.08. Median age was notably higher for HLA-B*5701 positive patients [32.7 (IQR: 28.3–41.3) years], compared to HLA-B*5701 negative patients [median: 31.6 (IQR: 26.8–38.3) years] (**p = 0.02)**. Predominant route of HIV acquisition was men who have sex with men(MSM) (54.3%, n = 987) followed by heterosexual transmissions (25.3% n = 461) and intravenous drug use (20.4% n = 371), with no differences in the distribution across HLA-B*5701 categories. Lymphocyte CD4 count at baseline was notably higher among HLA-B*5701 carriers (median 448 (294.5–662) cells/μl, vs. 352 (176–514) cells/μl for HLA-B*5701 negative), p<0.001, with lower number of individuals diagnosed with lymphocyte CD4<200 cells/μl [12.5% in the HLA-B*5701 positive vs 28.2% for the HLA-B*5701 negative, p<0.001]. Additionally, lower baseline viral load (IQR) associated with HLA-B*5701 [median: 4.2 (IQR: 3.5–4.8) log copies/ml vs 4.8 (IQR: 4.2–5.3) log copies/ml, for HLA-B*5701 positive and negative cases, respectively **(p<0.001)**.

### Association between HLA-B*5701 allele status, clinical characteristics and survival

Survival time was calculated for four timepoints (60, 120, 180 and 240 months corresponding to the 5, 10, 15 and 20 years), both for pretreatment period and time on cART. Because there were no cases of deaths among untreated patients with HLA-B*5701, this calculation was excluded. There were no notable differences in overall mortality between HLA-B*5701 positive and negative cases for any analyses **(Table 2)**.

**Table 2:** Overall survival rate for HLAB5701 positive and negative individuals.

death rate from diagnosis to end of observation							death rate for treated patients					
	HR	p (Cox regression)	lower 95% confidence interval	upper 95% confidence interval	% of patients who died during observation period (total no. of patients 2305) (Kaplan-Meier estimator) log-rank		HR	p (Cox regression)	lower 95% confidence interval	upper 95% confidence interval	% of patients who died during observation period (total no. of patients 2075) (Kaplan-Meier estimator) log-rank	
***survival for 60 months***												
HLA-B*5701 positive	0.5	p = 0.3	0.1	2.0	2 (1.5%)	p = 0.4	0.8	p = 0.7	0.2	2.4	3 (2.9%)	p = 0.7
HLA-B*5701 negative	ref.				71 (3.3%)		ref.				78 (4.0%)	
***survival for 120 months***												
HLA-B*5701 positive	0.6	p = 0.4	0.2	1.9	3 (2.3%)	p = 0.4	0.7	p = 0.5	0.2	2.1	3 (2.9%)	p = 0.5
HLA-B*5701 negative	ref.				93 (4.3%)		ref.				99 (5.0%)	
***survival for 180 months***												
HLA-B*5701 positive	0.5	p = 0.2	0.1	1.4	3 (2.3%)	p = 0.4	0.7	p = 0.5	0.3	1.9	4 (3.9%)	p = 0.5
HLA-B*5701 negative	ref.				125 (5.8%)		ref.				127 (6.5%)	
***survival for 240 months***												
HLA-B*5701 positive	0.5	p = 0.2	0.2	1.4	4 (3.1%)	p = 0.4	0.7	p = 0.5	0.3	1.9	4 (3.9%)	p = 0.5
HLA-B*5701 negative	ref.				140 (6.4%)		ref.				130 (6.6%)	

Because there were 0 cases of deaths among untreated patients with HLA-B*5701, this calculation was excluded.

When survival was analyzed across the 5,10,15 and 20 years timepoints of observation notable differences in the mortality rate were observed for the CD4 count characteristics (Table 3). Baseline CD4 under 200 cells/mm3 associated with higher mortality across every calculated timepoint, retaining significance even following 20 years of observation HR 2.5 (95%CI: 1.8–3.5, p<0,001)]. Survival probability was also divergent for the baseline viral load > 5 log copies/ml with higher mortality also seen for every observation timepoint with HR of 1.9 (95%CI: 1.3–2.9, p = 0.001)] for 20 year timepoint. Of note, differences in survival related to the transmission route were noted only for the 20 year timepoint with injection drug use associated with higher mortality [3.2% vs. 11.5% for sexual and IDU-related transmissions, HR 1.6 (95% CI: 1.0–2.5, p = 0.05)] **(Table 3)**.

**Table 3:** Baseline statistics and survival in examined cohort.

death rate for baseline statistics in examined cohort																								
	HR (60-months)	p (Cox regression)	lower 95% confidence interval	upper 95% confidence interval	% of patients who died during 60-months (Kaplan-Meier estimator) log-rank		HR (120-months)	p (Cox regression)	lower 95% confidence interval	upper 95% confidence interval	% of patients who died during 120-months (Kaplan-Meier estimator) log-rank		HR (180-months)	p (Cox regression)	lower 95% confidence interval	upper 95% confidence interval	% of patients who died during 180-months (Kaplan-Meier estimator) log-rank		HR (240-months)	p (Cox regression)	lower 95% confidence interval	upper 95% confidence interval	% of patients who died during 240-months (Kaplan-Meier estimator) log-rank	
***gender***																								
female	ref.	p = 0.3	0.7	2.6	11 (2.7%)	p = 0.3	ref.	p = 0.2	0.8	2.4	16 (3.9%)	p = 0.2	ref.	p = 0.1	0.9	2.2	23 (5.6%)	p = 0.1	**ref.**	**p = 0.06**	**1.0**	**2.4**	25 (6.1%)	**p = 0.03**
male	1.4				64 (3.3%)		1.4				82 (4.3%)		1.4				107 (5.6%)		**1.5**				121 (6.3%)	
**Route of transmition**																								
SEX	1.4	p = 0.4	0.6	3.2	35 (2.4%)	p = 0.4	ref.	p = 0.8	0.6	2.0	39 (2.7%)	p = 0.7	ref.	p = 0.1	0.9	2.3	44 (3.1%)	p = 0.1	**ref.**	**p = 0.05**	**1.0**	**2.5**	46 (3.2%)	**p = 0.02**
IDU	ref.				7 (2.0%)		1.1				16 (4.5%)		1.4				32 (8.9%)		**1.6**				41 (11.5%)	
**CD4 baseline**																								
<200 (cells/mm3)	**6.4**	**p<0.001**	**3.8**	**10.9**	48 (7.8%)	**p<0.001**	**3.7**	**p<0.001**	**2.4**	**5.7**	55 (9.0%)	**p<0.001**	**3.0**	**p<0.001**	**2.1**	**4.4**	70 (11.4%)	**p<0.001**	**2.5**	**p<0.001**	**1.8**	**3.5**	75 (12.3%)	**p<0.001**
>200 (cells/mm3)	**ref.**				19 (1.2%)		**ref.**				35 (2.1%)		**ref.**				49 (3.0%)		**ref.**				60 (3.7%)	
<500 (cells/mm3)	**6.0**	**p<0.001**	**2.2**	**16.5**	63 (3.9%)	**p<0.001**	**3.4**	**p<0.001**	**1.7**	**6.8**	81 (5.0%)	**p<0.001**	**2.8**	**p<0.001**	**1.6**	**4.8**	105 (6.4%)	**p<0.001**	**2.4**	**p<0.001**	**1.4**	**3.9**	117 (7.2%)	**p<0.001**
>500 (cells/mm3)	**ref.**				4 (0.7%)		**ref.**				9 (1.5%)		**ref.**				14 (2.3%)		**ref.**				18 (3.0%)	
**viral characteristics**																								
VL at diagnosis <100.000 copies/ml	**ref.**	**p<0.001**	**1.5**	**4.7**	19 (1.5%)	**p<0.001**	**ref.**	**p = 0.001**	**1.4**	**3.6**	28 (2.2%)	**p<0.001**	**ref.**	**p<0.001**	**1.4**	**3.2**	39 (3.0%)	**p<0.001**	**ref.**	**p = 0.001**	**1.3**	**2.9**	45 (3.5%)	**p = 0.001**
VL at diagnosis >100.000 copies/ml	**2.7**				33 (4.2%)		**2.2**				40 (5.1%)		**2.1**				51 (6.5%)		**1.9**				53 (6.7%)	

**Fig 1:**
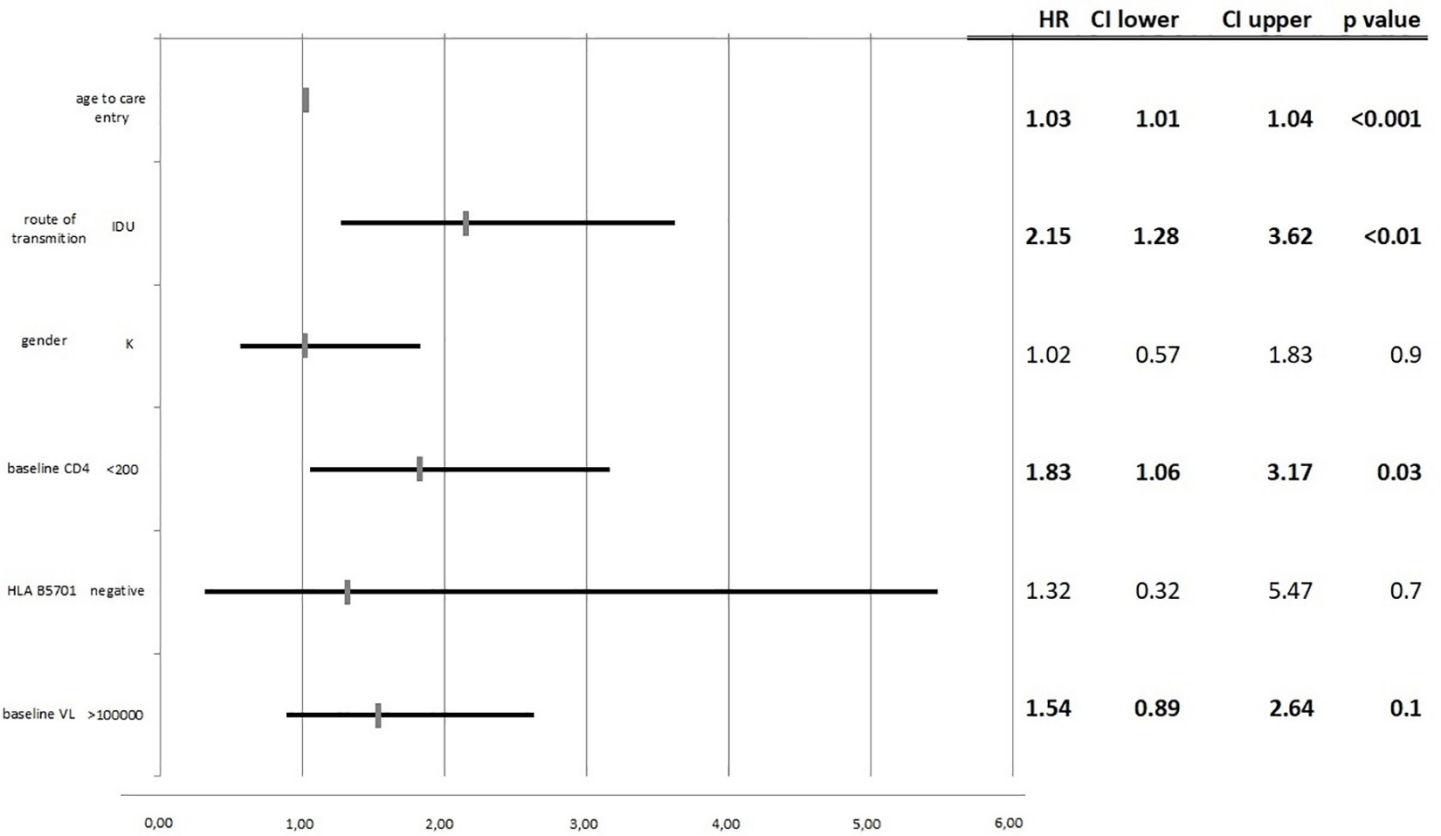
Multivariate logistic regression for the selected survival associated factors for the 20 year timepoint.

In multivariate proportional Cox hazards models, baseline CD4 <200 cells/μl, higher age and the IDU transmission route were associated with higher risk of mortality [HR: 1.83 (95%CI: 1.06–3.17, p = 0.03), HR: 1.03 (1.01–104, p<0.001) and HR: 2.15 (95%CI: 1.28–3.62, p<0.01)], respectively. Presence of HLA-B*5701 allele did not modify survival **(Fig 1)**.

## Discussion

This study investigates influence of HLA-B*5701 allele and clinical (age, gender, transmission category), virologic and immunologic characteristics (baseline HIV RNA viral load and lymphocyte CD4 counts) on 20 year survival in a large cohort of PWLH in Poland.

The frequency of HLA-B*5701 positive patients in examined cohort was 5.5% which is consistent with data of allele frequency in Caucasian population [9, 18, 19]. No significant influence on the survival was associated with the presence of the HLA-B*5701 variant, neither for the pre-cART and on-cART periods of observation. However, association of survival with an array of factors such as CD4 count, baseline viral load, route of transmission, and gender was found. Patients with HLA-B*5701 were included to cART in a smaller percentage 78,9% vs 96,59%, p<0,001, what is consistent with the number of viremic, and elite controllers in this group [20].

The data on life expectancy of people living with HIV in comparison with clinical characteristics in Polish population are limited, with only reports from Northern part of Poland comparing survival probability among patients with coinfection HIV/HCV [21]. Only few studies associated survival with HLA-B*5701 variant. A German study published in 2015, has indicated negative effect of the HLA-B*5701 presence on survival both among patients HIV positive with and without HCV coinfection with higher mortality associated with increased number of bacterial infections including sepsis, despite higher lymphocyte CD4 counts and lower baseline viral loads associated with this allele [22]. We did not confirm this observation in any models for the extended observation of 20 years.

It should be emphasized that one of the strongest predictors of increased risk of death among PLWH remains the lymphocyte CD4 count below 200 cells/ul. Numerous studies indicate that this is one of the key negative prognostic factors, especially if associated with high HIV-1 viral load prior to the cART introduction [23–26]. Our study confirmed those findings showing that lower pretreatment CD4 count and higher viral load could be associated to higher mortality among HIV positive individuals. Obviously, higher pretreatment viral loads or virologic failure during treatment increase risk of death and AIDS [27]. This was also observed in the current study with the negative effect of low CD4 counts (<200 cells/ul) and high viral loads (>5 log copies/ml) on survival observed for the long term across all the timepoints. Similar results were previously reported, with higher CD4 count and lower HIV viral load at baseline associated with increase all-cause mortality risk [28–30].

Moreover, transmission route, especially history of the injection drug use, may be a factor negatively affecting the survival–a result observed for the long term survival in our cohort. Survival studies on HIV-positive patient populations in the USA and Canada took into account biological traits such as race, gender, and alcohol and drug injection use. In the study by Keri N Althoff et al. the risk of death in the group of PWLH was analysed, with inclusion of race and gender as well as history of injection drug use. Life expectancy in Afro-American MSMs was shorter compared to the individuals of Caucasian ancestry, regardless the gender; also history of drug use associated with poorer survival, which is in line with our data [31]. A study by Samji H, Cescon A, Hogg RS, et al. investigated factors associated with decreased median life expectancy among PLWH compared to the general population [32]. The gap in life expectancy was lower for MSM compared to PWID, Caucasian vs. Afro-American ancestry or higher lymphocyte CD4 count. This result is also in accordance with our data, as among PWID or individuals with lower lymphocyte CD4 counts mortality rate was higher. Furthermore, African populations were examined for survival discrepancies related to gender and cART use [33, 34], observing a significant decline in HIV / AIDS-related mortality primarily among African women after initiation of antiretroviral therapy has been instituted, widening the gender-related survival gap. Result reported in our study was similar, with higher risk of mortality among men compared to women. Similar study, observing people living with HIV in Switzerland examined survival in the context of selected social factors such as education, family support, but also intravenous drug use and alcohol consumption [35]. It has been shown that the greatest differences in survival compared to the general population can be observed among the uneducated groups, while for highly educated people the difference in life expectancy compared to the general population was very small. It has also been shown that reducing cigarette smoking and rapid initiation of antiretroviral treatment can further extend the life expectancy of HIV-infected patients. Also in this study, higher risk of death associated with male gender, history injection drug use, and lower baseline CD4, which is consistent with our observations.

It needs to be mentioned that IDUs may be a surrogate marker of inadequate access to care. In fact this group of HIV patients was identified to be hard to reach population at each step of HIV continuum of care (https://pubmed.ncbi.nlm.nih.gov/30786803/).

In our study we have found no correlation between presence of HLA-B*5701 and survival probability in people living with HIV in Polish population, despite long term analysis and clearly observed influence of other factors such as age, gender, lymphocyte CD4 and HIV viral load. However, in our cohort of over two thousand PLWH we reported only four deaths among patients with HLA-B*5701 which accounts 3% mortality among the HLA-B*5701 allele carriers compared to 7.3% among HLA-B*5701 negative cases. Low mortality among patients with HLA-B*5701 may indicate the favorable profile related to this allele, however its low frequency may preclude from the detection of the association. As stated before, HLA-B*5701 is known to be associated with favorable CD4 count and viral load profiles at care entry which may influence the survival probability [36–38].

The analysis has the following key limitations. Firstly, mortality among the lost to follow-up individuals is unaccounted for, however it was impossible to generate these data. Secondly, due to the low HLA B*5701 prevalence the effect on survival may be difficult to capture, despite the fact that this observation was carried out in the large cohort of people who live with HIV observed for the prolonged period of time. Finally we did not investigate the influence of other genetic factors related to HIV such as HIV subtype or virus tropism (https://bmcinfectdis.biomedcentral.com/articles/10.1186/s12879-016-1480-8).

To sum up, in this study no signal related to the modification of survival probability associated with HLA B*5701 was detected, however we have confirmed a long-term influence of the baseline CD4 count and HIV viral load and transmission category on mortality among HIV-1 positive individuals. Genetic factors may add to the mortality risk, however this associations are often difficult to observe in effectively treated PWLH due to high efficacy of the antiretroviral regimens.

## Limitation of the study

In this study there was no calculation of survival of patients with detectable viral load against those with undetectable viral load as these long term treatment efficacy data were not collected Also, we did not collect the data on survival by the different causes of death, as, all case mortality was taken as end-point of the study.
