# Correction: Influence of HLA-B*5701 on 20 year survival rate among patients living with HIV

**DOI:** 10.1371/journal.pone.0272759

**Published:** 2022-08-03

**Authors:** Bogusz Jan Aksak-Wąs, Miłosz Parczewski, Anna Urbańska, Małgorzata Hackiewicz, Justyna D. Kowalska

The information listed in the Funding statement is incorrect. The correct Funding statement is as follows: This study was supported by the "Regional Initiative of Excellence" in 2019–2022 in the form of funds to MP [project no. 002 / RID / 2018/19].
